# Myricetin Attenuates IMQ-Induced Psoriatic Inflammation Through Multi-Target Modulation: Evidence from Network Pharmacology and Experimental Validation

**DOI:** 10.3390/ph18121802

**Published:** 2025-11-26

**Authors:** Deqiao Qin, Rongfen Gao, Lijuan Wu, Lingli Dong, Li Qin, Jingjiao Song

**Affiliations:** 1 Department of Rheumatology and Immunology, Tongji Hospital, Tongji Medical College, Huazhong University of Science and Technology, Wuhan 430022, China; d202382169@hust.edu.cn (D.Q.); gaorongfen@tjh.tjmu.edu.cn (R.G.); tjhdongll@163.com (L.D.); 2 Department of Dermatology, Traditional Chinese and Western Medicine Hospital of Wuhan, Tongji Medical College, Huazhong University of Science and Technology, Wuhan 430022, China; m202476901@hust.edu.cn; 3 Hubei Province & Key Laboratory of Skin Infection and Immunity, Wuhan 430022, China; 4 Experimental Medicine Center, Tongji Hospital, Tongji Medical College, Huazhong University of Science and Technology, Wuhan 430022, China

**Keywords:** myricetin, psoriasis, EGFR/AKT signaling, network pharmacology, natural flavonoid

## Abstract

**Background**: Psoriasis is a chronic inflammatory skin disease driven by keratinocyte hyperproliferation and immune dysregulation. Despite the availability of biologics and immunosuppressants, recurrence and adverse effects remain major limitations. Myricetin (Myr), a natural flavonoid with well-documented anti-inflammatory and immunomodulatory properties, has shown promise in inflammatory disorders; however, its efficacy and mechanisms in psoriasis have not been fully elucidated. **Methods**: The therapeutic effects of topical Myr (0.5–2%) were evaluated in an imiquimod (IMQ)-induced psoriatic mouse model. Network pharmacology and molecular docking were employed to predict potential targets, followed by validation using histological analysis, cytokine profiling, qPCR, and Western blotting. **Results**: Network analysis identified 52 overlapping targets between Myr and psoriasis, including TNF, PTGS2, MMP9, and EGFR, with enrichment in TNF, IL-17, and PI3K/AKT signaling pathways. Myr treatment significantly alleviated IMQ-induced erythema, scaling, and epidermal thickening, improved skin-barrier function, and reduced the expression of IL-6, IL-17A, and TNF-α. Molecular docking showed strong binding affinities of Myr with TNF, PTGS2, MMP9, and EGFR. Western blotting confirmed that Myr suppressed EGFR and AKT phosphorylation and downregulated *Mmp9*, *Ptgs2*, and *Tnf* expression. **Conclusions**: Myr exerts multi-target anti-psoriatic effects by inhibiting the EGFR/AKT axis and inflammatory mediators, highlighting its potential as a safe and effective natural therapeutic agent for psoriasis.

## 1. Introduction

Psoriasis is a chronic, inflammatory skin disorder characterized by the excessive proliferation of keratinocytes and immune system dysregulation [1]. Affecting approximately 2–3% of the global population, psoriasis significantly impacts patients’ quality of life [2]. While current treatments, including topical therapies, systemic immunosuppressants, and biologic agents, can provide symptomatic relief, they are frequently accompanied by disease recurrence after treatment withdrawal and notable side effects [3]. This highlights the need for safer, more effective therapeutic strategies for managing the disease.

Multiple inflammatory mediators and signaling pathways contribute to the pathophysiology of psoriasis, including tumor necrosis factor (TNF), prostaglandin–endoperoxide synthase 2 (PTGS2), and interleukin-17A (IL-17A) [4,5,6]. These proinflammatory factors can further induce the expression of matrix metalloproteinases (MMPs), such as MMP-9 and MMP-2, thereby promoting extracellular matrix degradation and skin tissue remodeling [7]. A vicious cycle between activated keratinocytes—which release cytokines and chemokines—and infiltrating immune cells perpetuates chronic inflammation and drives recurrent disease progression [8]. Among the key signaling pathways involved in psoriasis, the epidermal growth factor receptor (EGFR) and the PI3K-AKT pathway have been identified as critical regulators [9,10]. EGFR is notably overactivated in psoriatic lesions, contributing to epidermal hyperplasia and plaque formation, partly through crosstalk with the PI3K–AKT and mitogen-activated protein kinase (MAPK) cascades [11,12]. Interestingly, EGFR inhibitors used in cancer therapy have been reported to alleviate psoriatic symptoms, further underscoring the therapeutic relevance of this pathway in psoriasis management [12]. Consistently, a recent systematic review and network pharmacology analysis confirmed the pivotal roles of the PI3K/AKT/mTOR and JNK signaling pathways in psoriasis and identified EGFR, Sortilin, and Cyr61 as essential molecular hubs, suggesting that targeting these pathways may provide promising therapeutic benefits [13,14].

Myr, a natural flavonoid found in various fruits, vegetables, and medicinal plants, has attracted attention for its broad range of biological activities, including antioxidant, anti-inflammatory, and anticancer effects [15,16]. From a structural perspective, Myr bears a 3′,4′,5′-trihydroxylated (pyrogallol-type) B-ring [17]. This higher degree of B-ring hydroxylation has been associated with enhanced biological activities compared with less hydroxylated flavonols such as quercetin, kaempferol, or luteolin, and with increased binding affinity to serum albumin and certain protein targets, likely due in part to additional hydrogen-bonding interactions [18,19]. Recent studies have shown that Myr exerts anti-inflammatory properties by modulating key signaling pathways involved in immune responses, including inhibiting the production of pro-inflammatory cytokines such as IL-6, IL-1β, and TNF-α, which are central to psoriasis pathogenesis [20,21]. In dermatological research, Myr has demonstrated therapeutic potential in a calcipotriol-induced atopic dermatitis (AD) mouse model, where it significantly alleviated inflammatory symptoms through its anti-inflammatory and antiallergic functions [20,22]. Moreover, Previous studies indicate that Myr can engage the EGFR/PI3K/AKT axis in relevant models, including reports of EGFR downregulation and suppression of PI3K/AKT–mTOR signaling in mechanistic overviews [23,24,25]. In human keratinocytes, Myr suppresses TNF-α–induced AKT/mTOR/NF-κB activation and reduces inflammatory mediator production [26], and it promotes TRPV4-mediated differentiation while enhancing barrier-related proteins such as keratin 10, filaggrin and involucrin [27]. Collectively, these mechanistic, cellular, and structural features support Myr as a rational lead for EGFR-related multi-target modulation in psoriasis. However, to date, the therapeutic potential and molecular mechanisms of Myr in psoriasis have not been elucidated.

In this study, we evaluated the therapeutic effects of Myr on IMQ-induced psoriatic inflammation in a mouse model. Using network pharmacology, we identified 52 overlapping genes between the predicted targets of Myr and psoriasis-related molecules. Further analysis revealed that *MMP9*, *TNF*, *PTGS2*, and *EGFR* were among the major potential targets of Myr. Moreover, Gene Ontology (GO) and Kyoto Encyclopedia of Genes and Genomes (KEGG) pathway enrichment analyses showed that, in addition to the TNF and IL-17 signaling pathways, the PI3K/AKT signaling pathway was the most significantly enriched, suggesting a potential therapeutic role of Myr in psoriasis. In vivo experiments further validated its efficacy, showing that Myr significantly alleviated skin inflammation, reduced epidermal hyperplasia, and decreased inflammatory cell infiltration. Myr also suppressed the expression of pro-inflammatory cytokines, including IL-17 and TNF-α. Consistently, molecular docking analysis demonstrated strong binding affinities of Myr with MMP9, TNF, PTGS2, and EGFR. Western blotting further verified that Myr significantly inhibited the activation of the EGFR and AKT signaling pathways, accompanied by downregulation of *Mmp9*, *Tnf*, and *Ptgs2* expression. These findings provide strong evidence that Myr effectively mitigates psoriatic inflammation, positioning it as a promising natural therapeutic agent for psoriasis.

## 2. Results

### 2.1. Network Pharmacology and Functional Analysis of Myr Targets in Psoriasis

**Figure 1:**
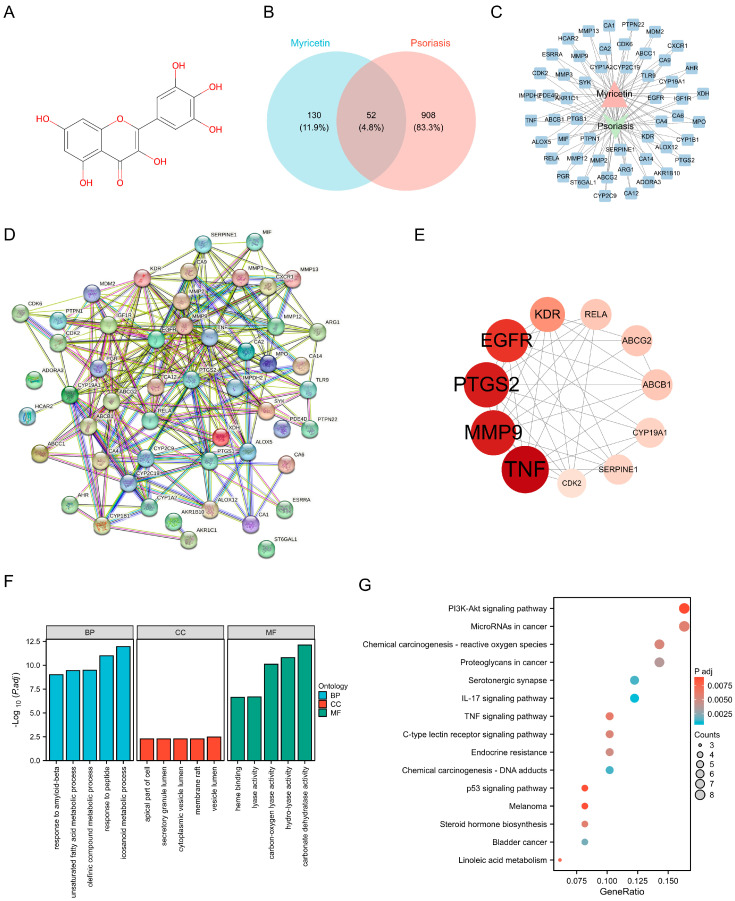
Network Pharmacology reveals the mechanism of Myr in the treatment of psoriasis. (**A**) Chemical Structure of Myr. (**B**) Venn diagram of Myr and psoriasis shared targets. (**C**) Visualization of the 52 Shared Target. (**D**) Protein−protein interaction networks in the PPI network. (**E**) Top 11 protein−protein interaction networks. The larger the circle area and the darker the color, the higher the degree value. (**F**) GO enrichment analysis of common targets. (**G**) KEGG enrichment analysis of common targets (top 15, *p* < 0.01).

Through analysis using the Swiss Target Prediction and TargetNet databases, 182 potential targets for Myr were identified following prediction, curation, and de-duplication (The chemical structure of Myr is shown in Figure 1A). Additionally, 960 psoriasis-related targets were retrieved and screened from the DrugBank, GeneCards, and TTD databases. By comparing these psoriasis-related targets with those of Myr, 52 common targets for Myr’s therapeutic effect on psoriasis were identified. The Venn diagram of these intersection targets is shown in Figure 1B. A “Myr-Active Ingredients-Intersection Targets” network was constructed using Cytoscape 3.10.0, consisting of 52 nodes and 1173 interaction relationships (Figure 1C). The network shows the relationships between Myr, its active ingredients, and the disease targets. To further explore these interactions, a protein–protein interaction (PPI) network was created using data from the STRING database, and key targets were selected with the Centiscape 2.2 plugin. The PPI network for the common targets of Myr in psoriasis contains 49 nodes and 246 interaction relationships, with 11 key targets identified (Figure 1D). Among these, the top-ranking targets by degree value included TNF, MMP9, PTGS2, and EGFR (Figure 1E). GO functional enrichment analysis revealed strong associations with biological processes such as response to amyloid-beta, unsaturated fatty acid metabolic process, response to peptide (Figure 1F). The common targets were also linked to various cellular components and molecular functions, such as vesicle lumen, apical part of cell, and carbon–oxygen lyase activity (Figure 1F). Additionally, KEGG pathway enrichment analysis highlighted 88 enriched pathways, including the PI3K-AKT signaling pathway, MicroRNAs in cancer, IL-17 signaling pathway, and TNF signaling pathway (Figure 1G).

Overall, the results suggest that Myr may exert therapeutic effects on psoriasis through multi-target and multi-pathway mechanisms.

### 2.2. Myr Alleviates IMQ-Induced Psoriatic Skin Phenotype

**Figure 2:**
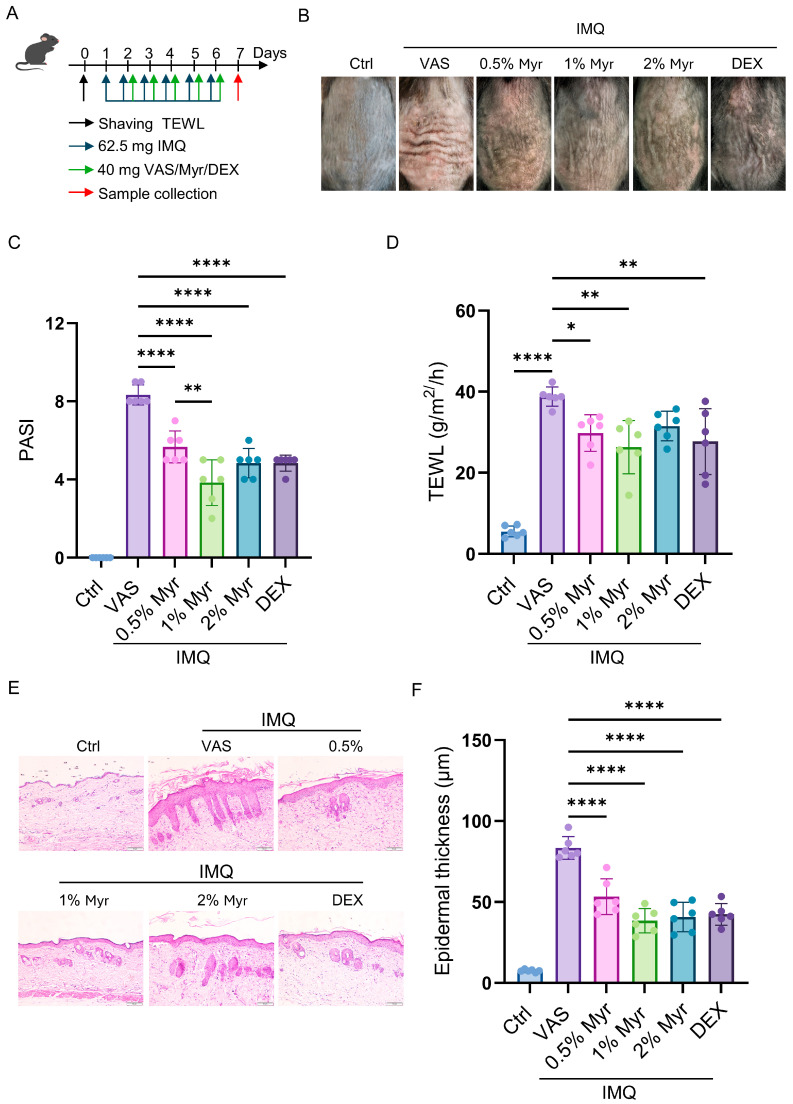
Myr alleviates IMQ-induced psoriatic skin phenotype. (**A**) Schematic illustration of the strategy for inducing psoriasis-like skin inflammation with IMQ and subsequent treatment with Myr ointment in WT mice. (**B**) Representative photos of mouse back skin. (**C**) Scoring curves of back skin thickness, scaling, and erythema (PASI). (**D**) The TEWL values. (**E**) HE staining of skin sections. Scale bar = 50 μm. (**F**) The average thickness of epidermis. *n* = 6 biological samples from two independent experiments. Data are shown as mean ± SD. For (**C**,**D**,**F**), one way ANOVA with Tukey’s multiple comparisons test. * *p* < 0.05, ** *p* < 0.01, **** *p* < 0.0001.

We further investigated the therapeutic effect of Myr on IMQ-induced psoriatic-like inflammation (Figure 2A). The results demonstrated that Myr significantly alleviated erythema, scales, and skin thickening induced by IMQ, with the most pronounced therapeutic effects observed at a 1% concentration (Figure 2B,C). Additionally, previous studies have highlighted that skin-barrier dysfunction is a key feature of psoriasis and plays a major role in its onset and exacerbation [28]. To assess the impact on skin-barrier function, we measured transepidermal water loss (TEWL). The results revealed a significant increase in TEWL in IMQ-treated mice, while Myr treatment markedly improved the skin-barrier function (Figure 2D). Further histological analysis showed that Myr effectively reduced hyperproliferation of keratinocytes and significantly improved epidermal thickening, particularly at concentrations of 1% and 2% (Figure 2E,F). In summary, Myr effectively alleviates IMQ-induced psoriatic inflammation by enhancing skin-barrier function and reducing epidermal thickening.

### 2.3. Myr Alleviates Keratinocyte-Dominant Inflammatory Responses in the Skin of IMQ-Induced Mice

**Figure 3:**
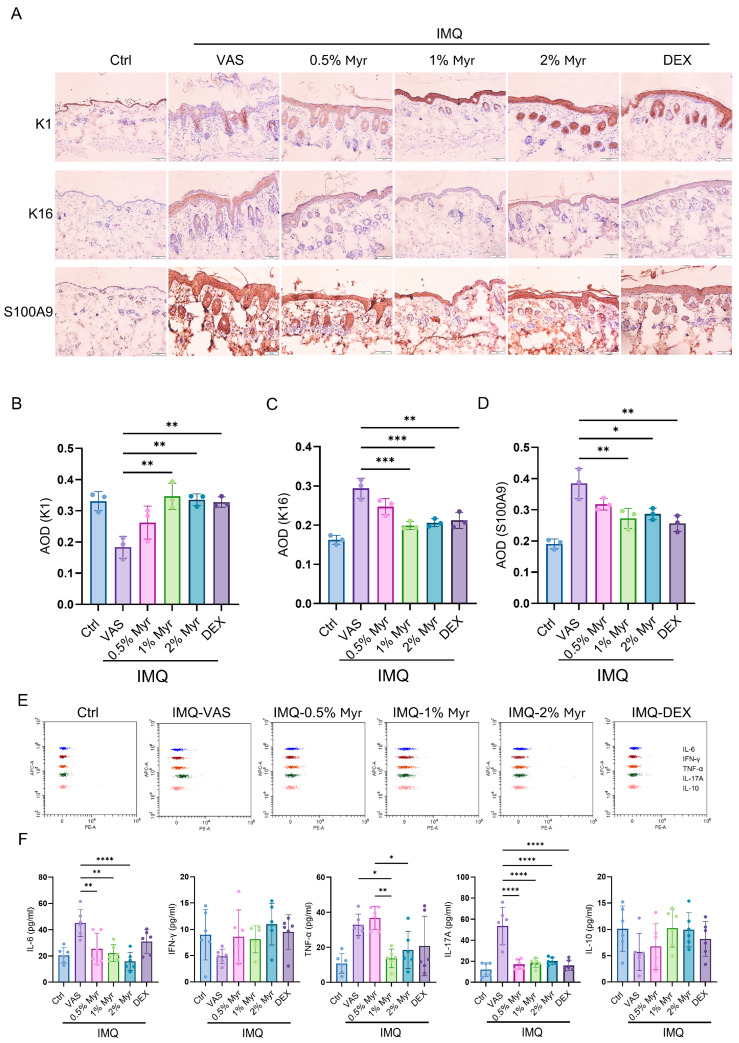
Myr alleviates keratinocyte-dominant inflammatory responses in the skin of IMQ-induced mice. (**A**) IHC staining for K1, K16 and S100A9. *n* = 3, Scale bar = 50 μm. (**B**–**D**) AOD of K1, K16 and S100A9, respectively. (**E**,**F**) CBA was used to detect cytokine secretion in the supernatant of lysed skin tissue. *n* = 6 biological samples from two independent experiments. Data are shown as mean ± SD. For (**B**–**D**,**F**), one way ANOVA with Tukey’s multiple comparisons test. * *p* < 0.05, ** *p* < 0.01, *** *p* < 0.001, **** *p* < 0.0001.

Psoriasis is characterized by excessive proliferation and abnormal differentiation of keratinocytes, leading to skin thickening and scaling [8]. This process is closely associated with abnormal immune activation, particularly involving T cells and cytokines, which further exacerbate local inflammation. We first performed immunohistochemistry to examine markers of keratinocyte differentiation (K1), proliferation (K16), and antimicrobial peptide secretion (S100A9). The results showed that K1 expression was significantly reduced after IMQ induction, whereas treatment with 1% and 2% Myr markedly restored K1 levels, suggesting that Myr can improve abnormal keratinocyte differentiation (Figure 3A,B). In addition, Myr treatment at 1% and 2% concentrations significantly decreased the IMQ-induced expression of K16, indicating its ability to suppress keratinocyte hyperproliferation (Figure 3A,C). At similar concentrations, Myr also notably reduced the secretion of S100A9 (Figure 3A,D). Cytokine secretion in the skin was evaluated using CBA, which showed that IMQ induction markedly increased IL-6, TNF-α, and IL-17A levels. Treatment with 1% and 2% Myr, as well as dexamethasone (DEX), significantly reduced the IMQ-induced elevation of IL-6 and IL-17A. Notably, 1% Myr also suppressed TNF-α release, with an effect even superior to that of DEX (Figure 3E,F). Collectively, these findings suggest that Myr holds promising potential in restoring inflammatory balance in psoriatic skin.

### 2.4. Myr Attenuates Systemic Immune Responses in IMQ-Induced Psoriatic Mice

**Figure 4:**
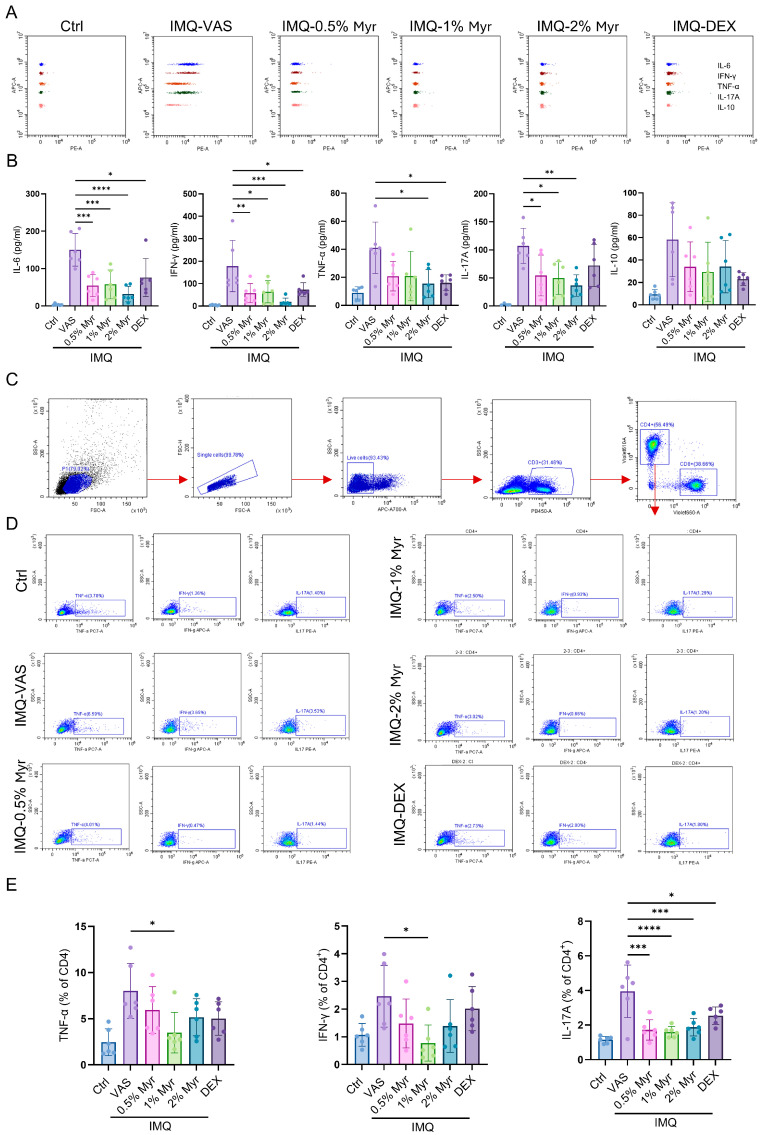
Myr attenuates systemic immune responses in IMQ-induced psoriatic mice. (**A**,**B**) CBA was used to detect inflammatory cytokine release in the serum of IMQ-induced mice. (**C**,**D**) Gating strategy of flow cytometry analysis in the splenocytes of mice. (**E**) Flow cytometry analysis of IL-17A, IFN-γ or TNF-α producing CD4^+^ T cells in the splenocytes. *n* = 6 biological samples from two independent experiments. Data are shown as mean ± SD. For (**B**,**E**), one way ANOVA with Tukey’s multiple comparisons test. * *p* < 0.05, ** *p* < 0.01, *** *p* < 0.001, **** *p* < 0.0001.

Psoriasis is a chronic inflammatory disease characterized not only by skin lesions but also by systemic immune dysregulation. Aberrant T cell-mediated responses and cytokine release (e.g., IL-17, IFN-γ, TNF-α) drive keratinocyte hyperproliferation and differentiation abnormalities while also affecting cardiovascular and metabolic systems, underscoring psoriasis as a systemic immune-mediated disorder [29]. We assessed inflammatory cytokine release in the serum of IMQ-induced mice using CBA. The results showed that IL-6, IFN-γ, TNF-α, and IL-17A were significantly elevated after IMQ induction (Figure 4A,B). Treatment with Myr markedly reduced these cytokines, particularly at concentrations of 1% and 2% (Figure 4A,B). Splenic profiling showed that IMQ induction markedly increased the frequencies of CD4^+^ T cells producing TNF-α, IFN-γ, and IL-17A. Treatment with Myr at all tested concentrations, as well as with DEX, significantly reduced the proportion of IL-17A^+^ CD4^+^ T cells. Notably, only 1% Myr treatment also led to a significant reduction in TNF-α^+^ and IFN-γ^+^ CD4^+^ T cells, underscoring its superior immunomodulatory activity (Figure 4C–E). These results highlight the potential of Myr to restore immune balance and attenuate systemic inflammatory responses in psoriasis.

### 2.5. Myr Attenuates Imiquimod-Induced Skin Inflammation in Psoriasis-like Mice Accompanied by Inhibiting the EGFR/AKT Signaling Pathway and Decreasing the Levels of Mmp9, Ptgs2 and Tnf

**Figure 5:**
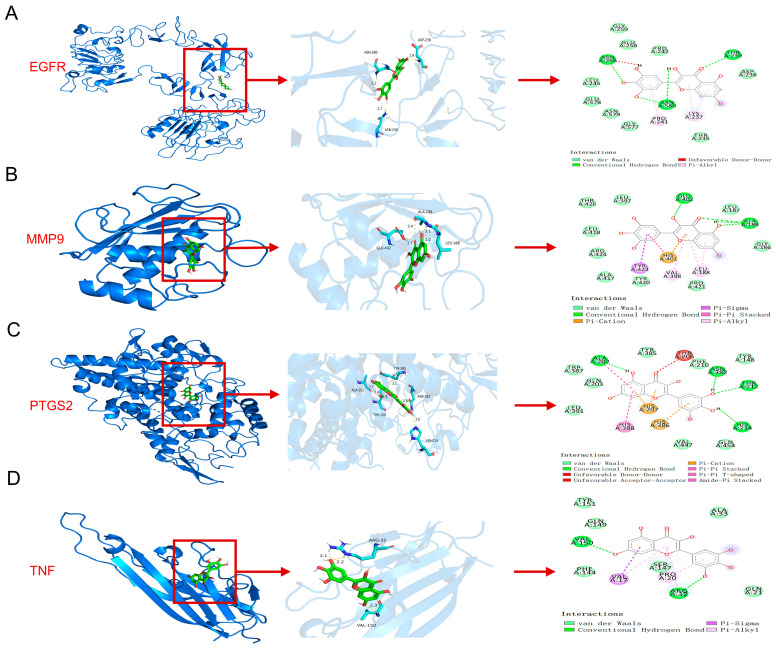

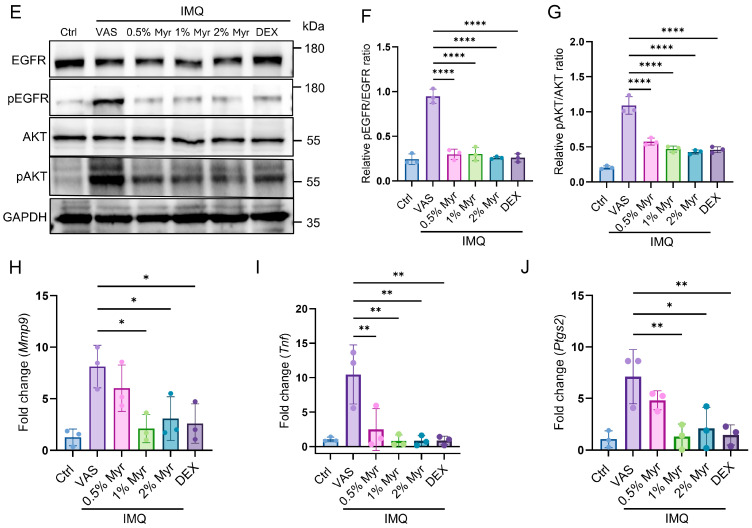
Myr attenuates imiquimod-induced skin inflammation in psoriasis-like mice accompanied by inhibiting the EGFR/AKT signaling pathway and decreased the level of *Mmp9*, *Ptgs2* and *Tnf*. (**A**–**D**) Protein–ligand docking reveals a stable protein–ligand interaction between Myr and TNF, MMP9, PTGS2, as well as EGFR. (**E**) Western blot analysis of EGFR and AKT in IMQ-induced mouse model. (**F**,**G**) Quantitative analysis of Western blot results for Figure 5E. (**H**–**J**) The mRNA levels of *Mmp9*, *Tnf*, and *Ptgs2* in the skin were determined by RT-qPCR. Data are shown as mean ± SD. For (**F**–**J**), one way ANOVA with Tukey’s multiple comparisons test. * *p* < 0.05, ** *p* < 0.01, **** *p* < 0.0001.

Further molecular docking was performed between Myr, and the top four proteins identified in Figure 1E (TNF, MMP9, PTGS2, and EGFR). Protein–ligand docking analysis using AutoDock Vina generated complex models of Myr with each of these proteins, with predicted binding free energies all lower than −4 kcal/mol, indicating stable interactions between Myr and TNF, MMP9, PTGS2, as well as EGFR (Figure 5A–D) [30]. Structural analysis further revealed that, although the interface areas between Myr and the proteins were relatively small—likely due to the compact nature of their overall structures—multiple interaction sites were present within the limited contact surfaces. Surface representation models demonstrated that Myr and the target proteins achieved close surface complementarity and formed stable complexes through several hydrogen bonds, which contributed substantially to complex stability (Figure 5A–D). These findings suggest that Myr forms stable complexes with TNF, MMP9, PTGS2, and EGFR through multiple surface-mediated hydrogen bonds, exhibiting strong binding affinity and structural stability—characteristics typical of high-affinity molecular interactions.

Based on the molecular docking results of EGFR with Myr and the functional analysis of potential targets, the PI3K/AKT signaling pathway was identified as the most significantly enriched (Figure 1G). To further validate this finding, proteins extracted from mouse skin were analyzed by Western blot. The results demonstrated that IMQ induction markedly elevated the phosphorylation levels of EGFR and AKT, whereas Myr treatment effectively reversed these changes (Figure 5E–G). In addition, RT-qPCR analysis of mouse skin revealed that 1% and 2% Myr significantly attenuated the IMQ-induced upregulation of *Mmp9* and *Ptgs2*. Moreover, Myr treatment suppressed *Tnf* expression at all tested concentrations (Figure 5H–J).

In summary, Myr stably binds TNF, MMP9, PTGS2, and EGFR, and functionally suppresses IMQ-induced EGFR/AKT activation and downstream inflammatory mediators.

## 3. Discussion

**Figure 6:**
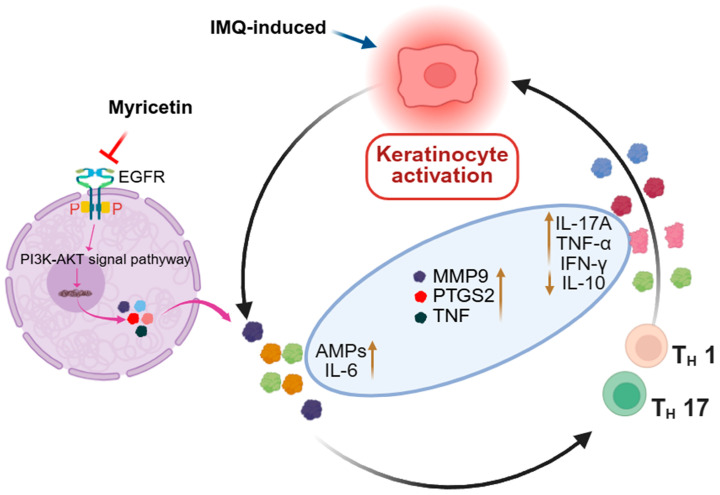
The schematic diagram shows the effect and mechanism of Myr alleviates IMQ-induced psoriatic inflammation by inhibiting the EGFR/AKT Signaling Pathway.

This study provides evidence that Myr exerts anti-psoriatic activity through multi-target and multi-pathway mechanisms, prominently involving inhibition of the EGFR/AKT axis and suppression of inflammatory mediators (Figure 6). These findings align with the established roles of epidermal hyperproliferation and aberrant immune responses in psoriasis pathogenesis, while extending current knowledge by demonstrating that Myr may directly modulate receptor tyrosine kinase-mediated signaling involved in disease progression.

Previous studies have shown that Myr suppresses the expression of multiple inflammatory cytokines and inhibits the activation of key inflammatory signaling pathways [31]. Consistent with these findings, our results further demonstrated that Myr markedly attenuated both local and systemic inflammation in psoriatic mice. Myr was predicted to interact with TNF, PTGS2, and MMP9—targets well documented to contribute to lesion persistence, extracellular matrix remodeling, and inflammatory amplification in psoriasis [4,5,32]. Prior studies have shown that elevated MMP9 promotes tissue damage and immune infiltration, while PTGS2-derived prostaglandins exacerbate cutaneous inflammation [33]. By affecting these nodes simultaneously, Myr appears to act in a network-oriented manner, contrasting with single-target biologics. This multi-level regulation may underlie its capacity to rebalance epidermal and immune responses.

In addition to its well-known anti-inflammatory properties, this study revealed that Myr exerts therapeutic effects on psoriasis by modulating the activity of EGFR, a pivotal receptor tyrosine kinase regulating epidermal homeostasis [34,35]. EGFR signaling has long been implicated in keratinocyte proliferation and the amplification of psoriatic inflammation [36], yet therapeutic strategies have largely centered on cytokine blockade (e.g., TNF-α or IL-17 inhibitors) rather than targeting receptor pathways directly [3]. Previous work has shown overexpression and activation of EGFR in psoriatic lesions and its cross-talk with IL-17A–driven responses [37], while PI3K/AKT/mTOR signaling has been recognized as a central regulator of epidermal growth and metabolism [38]. Our findings indicate that Myr-mediated inhibition of the EGFR/PI3K/AKT signaling pathway is mechanistically linked to immune regulation rather than simple immune suppression, as it attenuates EGFR/AKT-driven keratinocyte hyperactivation and reduces epidermal-derived inflammatory signaling. This modulation contributes to normalization of dysregulated immune responses and restore immune balance in the skin microenvironment. Notably, this mechanism complements earlier reports on flavonoids such as quercetin and kaempferol, which mainly act through NF-κB or MAPK inhibition [39,40,41]. Consistently, the regulatory effect of Myr on EGFR has also been observed in herpes simplex virus (HSV) infection, and this modulation is closely associated with the PI3K/AKT signaling pathway [42]. The potential of Myr to modulate EGFR activity therefore represents a mechanistic distinction and could broaden the therapeutic landscape beyond cytokine-centered interventions. From a human health perspective, its multi-target, low-toxicity pharmacological profile highlights Myr’s potential as a safe and effective candidate for managing immune-mediated cutaneous disorders. Future investigations involving human keratinocyte–immune co-culture models, together with clinical pharmacokinetic and safety evaluations, will be essential to confirm its translational applicability and therapeutic benefits in humans.

Notably, the therapeutic efficacy of Myr did not increase proportionally with its concentration. Specifically, 1% Myr showed the most pronounced improvement in psoriatic symptoms, whereas 2% Myr was slightly less effective. This non-linear dose–response trend may be explained by several formulation-related factors. First, at higher drug loading, solubility and diffusion limitations may occur due to local supersaturation, microcrystal formation, or increased matrix viscosity, thereby reducing the effective release rate and transdermal flux. Second, surface saturation and altered drug distribution could take place at the stratum corneum, where excessive deposition of Myr forms a “reservoir,” diminishing the penetration gradient and decreasing the fraction of free drug that reaches viable skin layers. Importantly, since all formulations contained an equal amount of solvent, the observed difference is unlikely to be caused by vehicle effects. In future studies, we plan to explore the use of hydrogels, lipid-based nanocarriers, and cyclodextrin or polymer complexes to optimize the formulation and dosing window of Myr, thereby enhancing its therapeutic efficacy and skin compatibility.

Furthermore, the comparison with corticosteroid therapy is also noteworthy. While glucocorticoids remain a mainstay in psoriasis management, their long-term use is limited by systemic toxicity [43,44]. Natural compounds such as flavonoids have been proposed as safer alternatives, yet many lack sufficient potency [45]. Prior studies reported mainly modest effects of flavonoids in skin inflammation, whereas the current findings suggest Myr exerts broader immunomodulation, including suppression of Th1/Th17-associated cytokines. This raises the possibility that Myr, alone or in combination, could reduce reliance on systemic immunosuppressants.

Despite these promising findings, several key issues remain. The IMQ model reflects acute rather than chronic psoriasis, limiting its translational relevance. In future studies, chronic or repeated IMQ challenge models will be employed to better mimic sustained psoriatic inflammation and to evaluate the pharmacokinetic and pharmacodynamic profiles of Myr under prolonged treatment conditions. Molecular docking and pathway analyses require further validation through direct binding and genetic studies. Moreover, the pharmacokinetics, skin absorption, and long-term safety of Myr are not well characterized. Its poor water solubility and low skin permeability also hinder clinical application. Although solvents such as DMSO were necessary in this study to dissolve Myr, they are known to potentially irritate the skin or alter barrier integrity. To overcome these limitations, strategies such as nano-formulations—for example, HPBCD/PVP-based MyNF systems—have shown improved activity in UVB-induced keratinocyte damage models [34]. Although a relatively low toxicity profile [42], extensive exploratory work is needed to confirm target engagement, optimizing formulations for skin delivery, and testing efficacy in chronic psoriasis models. Therefore, further research will focus on developing less-irritating and more biocompatible delivery systems, such as hydrogels, lipid-based nanocarriers, and cyclodextrin or polymer complexes, to improve both the solubility and topical safety profile of Myr formulations.

## 4. Materials and Methods

### 4.1. Ethics Statement

All experimental procedures involving animals were approved by the Institutional Animal Care and Use Committee of Huazhong University of Science and Technology and adhered to the guidelines set forth in the Guide for the Care and Use of Laboratory Animals. The animal protocol (No. TJH-202009001) was approved on 18 December 2020.

### 4.2. Preparation of Myr

Myr (CAS# HY-15097, purity ≥ 98%) was purchased from MedChemexpress (MCE, Shanghai, China). Topical ointments containing 0.5%, 1%, and 2% Myr were prepared. Briefly, Myr was first dissolved in a basal solution consisting of 10% DMSO, 40% PEG300, 5% Tween-80, and 45% saline to yield a clear solution (solubility ≥ 2.08 mg/mL, 6.54 mM). The resulting solution was then thoroughly mixed with white petrolatum (Vaseline, Englewood Cliffs, NJ, USA) to prepare the ointment base at the indicated concentrations.

### 4.3. Animal Experiment

WT C57BL/6J mice were housed in specific-pathogen-free conditions at the Laboratory Animal Experiment Center of Tongji Hospital, Tongji Medical College, Huazhong University of Science and Technology. The feed, bedding, cages, and drinking water were all sterilized. The room temperature was maintained at approximately 25 °C with a relative humidity of 50%, and a 12 h light/dark cycle was used.

To establish the psoriasis mouse model, 62.5 mg of 5% IMQ cream (Aldara, 3M Pharmaceuticals, Northridge, CA, USA) was topically applied to shaved 2.5 cm × 2.5 cm areas of the back skin of the mice daily for six consecutive days. The severity of skin lesions was assessed daily using the Psoriasis Area and Severity Index (PASI), evaluating skin thickness (wrinkles), scaling, and erythema. Skin wrinkles, scaling, and erythema were scored from 0 to 4: 0, none; 1, mild; 2, moderate; 3, severe; and 4, very severe. On day 6.5, mice were euthanized, and samples were collected for subsequent experiments.

After the second IMQ induction, IMQ-treated mice were randomly divided into five groups (IMQ + vehicle, IMQ + Myr 0.5%, IMQ + Myr 1%, IMQ + Myr 2%, and IMQ + DEX; *n* = 6 per group). A separate healthy control group received only vehicle without IMQ treatment (*n* = 6).

To ensure that the total amount of solvent was consistent among all treatment groups, the same basal solvent described in Section 4.2 was additionally supplemented to the VAS, 0.5%, and 1% Myr groups, since the 0.5% and 1% formulations originally contained slightly less solvent than the 2% Myr ointment. This adjustment ensured that each group received an equal volume of solvent components during topical administration.

Mice were then topically treated once daily in parallel with IMQ application. Each mouse received 40 mg of 0.5%, 1%, or 2% Myr ointment, Vaseline (vehicle control), or DEX cream (positive control) per application until the end of the modeling period. On day 6.5 after treatment initiation, the mice were euthanized, and dorsal skin tissues were collected for subsequent histological and molecular analyses.

All assessments, including PASI scoring, histopathological examination, and immunohistochemical quantification, were performed by investigators blinded to group allocation to minimize bias. The study design ensured consistent solvent exposure, clear group differentiation, randomization, and reproducibility across replicates.

### 4.4. Preparation of Mouse Skin Single-Cell Suspension

Mice were sacrificed at the indicated time points, and their back skin was separated. The skin was cut into small pieces and incubated in RPMI 1640 medium containing 3.5 mg/mL collagenase I (Biosharp, Beijing, China; #BS163), 0.02 mg/mL DNase I (Biosharp, #BS137), and 0.002 mg/mL hyaluronidase (Biosharp, #BS171) at 37 °C for 60 min. The digested skin pieces were passed through a 70-μm nylon mesh, and fresh RPMI 1640 was added to the suspensions to neutralize the enzyme activity. The suspensions were then centrifuged at 1400 rpm for 10 min, and the pellets were resuspended in PBS (1% BSA) and counted.

### 4.5. Protein–Ligand Docking Using AutoDock Vina

Molecular docking was performed using AutoDock Vina 1.5.6 to evaluate the interaction between Myr and target proteins. The conformation showing the highest frequency and best binding affinity was selected as the optimal result, and the complexes were visualized using PyMOL 3.1 and Discovery Studio 2019. Semi-flexible docking was employed, allowing ligand flexibility while keeping the protein rigid. The spatial complementarity between the ligand and the protein surface suggested favorable binding. Key amino acid residues at the binding site and hydrogen bond formation were analyzed, with hydrogen bond lengths of 1.5–3.5 Å considered optimal for stable interactions. The binding energy (ΔG) was used as the main evaluation criterion, and values below −4.0 kcal/mol indicated strong and stable binding.

### 4.6. Surface and Intracellular Staining of Splenocytes and Skin Single Cells

For splenocyte preparation, about 1 × 10^6^ cells were stimulated and blocked with Cell Activation Cocktail (Biolegend, San Diego, CA, USA; #423303), followed by surface antibody staining with CD3 (BV421, BD Biosciences, Franklin Lakes, NJ, USA, #563024), CD4 (BV605, BD Biosciences, #563151), and CD8 (BV650, BD Biosciences, #563234). Cells were then fixed and permeabilized, and intracellular cytokine staining was performed overnight at 4 °C using antibodies for IL-17A (PE, BD Biosciences, #559502), TNF-α (PE-Cy7, BD Biosciences, #560658), and IFN-γ (APC, BD Biosciences, #554413). Cells were stained with fixable viability stain (FVS) to exclude non-viable cells and gated to exclude doublets.

All data were acquired and analyzed using a CytoFLEX flow cytometer (Beckman Coulter, Brea, CA, USA) and FlowJo software (version V10).

### 4.7. Cytometric Bead Array (CBA)

Sera were collected from mice at various time points. IL-6, IL-17A, TNF-α, IFN-γ and IL-10 cytokines were measured simultaneously using the mouse Th1/Th2/Th17 CBA kit (BD Biosciences, 560485) according to the manufacturer’s instructions. For skin cytokine detection, 200 μL of PBS containing a protease inhibitor mixture was added to 20 mg of mouse skin and homogenized in a frozen grinder (JXFSTPRP-CLN-48, Shanghai Jingxin, Shanghai, China). Supernatants were collected after centrifugation at 14,000 rpm for 30 min at 4 °C. Samples were analyzed using CytoFLEX-13 (Beckman Coulter). Data were analyzed using FCAP Array software (1.0.19.0).

### 4.8. RNA Extraction and Quantitative Real-Time PCR (qPCR)

Total RNA was isolated from skin, cell lines, or spleens using the RNA Kit (Omega Bio-tek, Norcross, GA, USA) following the manufacturer’s instructions. RNA was reverse-transcribed into cDNA using HiScript III RT SuperMix for qPCR (Vazyme Biotech Co., Ltd., Nanjing, China) and gDNA Clean for qPCR, and qRT-PCR was performed with a real-time system (SYBR Green). Gene expression was calculated using the 2^−ΔΔCT^ method relative to the housekeeping gene GAPDH. All primers were synthesized by Quintara Corporation (Wuhan, China).

### 4.9. Western Blot

Proteins were extracted from cell lysates, separated by 10% SDS-PAGE, and analyzed by Western blot. Protein bands were visualized using chemiluminescence and analyzed with Image J software (1.53t), with band intensities normalized to GAPDH.

### 4.10. Histological and Immunohistochemical (IHC) Analysis

Skin tissues were fixed in 10% formalin, embedded in paraffin, and stained with hematoxylin and eosin (HE). Images were obtained using an upright microscope (Olympus, Tokyo, Japan; BX53). Three random fields were selected from each mouse, and the average epidermal thickness was calculated by two blinded observers using Image J software.

For IHC, deparaffinized sections were treated with 3% hydrogen peroxide to block endogenous peroxidase activity, followed by antigen retrieval by boiling in 10 mM sodium citrate buffer (pH 6.0) for 15 min. Sections were blocked with 10% sheep serum and incubated with the primary antibodies overnight: K1 (Invitrogen, Waltham, MA, USA; PA5-114755, 1:400 dilution), K16 (Invitrogen, PA5-99490, 1:600 dilution), LL37 (Abcam, Cambridge, UK; ab180760, 1:400 dilution), and S100A9 (Boster, Jincheon, Republic of Korea; PB0718, 1:500 dilution). Secondary antibody conjugated to horseradish peroxidase was used, and images were captured using an upright microscope (Olympus BX53). Image J software was used for image analysis.

### 4.11. Biophysical Skin Measurements (TEWL)

Trans-epidermal water loss (TEWL) was measured from the dorsal skin of mice using the VAPOSCAN AS-VT100RS machine (Asahi Biomed, Tokyo, Japan). All measurements were performed three times per mouse. TEWL was recorded at an ambient temperature of about 25 °C and humidity of about 50%.

### 4.12. Network Pharmacology

Network pharmacology was applied to identify the active ingredients and their targets of Myr, as well as the potential psoriasis-related targets. First, the SMILES number of Myr was obtained from the PubChem database (https://pubchem.ncbi.nlm.nih.gov/, accessed on 10 September 2025), and this information was used to query the Swiss Target Prediction database (http://swisstargetprediction.ch/, accessed on 10 September 2025) and TargetNet database to collect the active targets of Myr. Psoriasis-related targets were then retrieved from several databases, including GeneCards (https://www.genecards.org/, accessed on 10 September 2025), DrugBank (https://go.drugbank.com/, accessed on 10 September 2025), and the TTD database (https://ttd.idrblab.cn/, accessed on 10 September 2025) using “psoriasis” as a keyword. To identify the intersection targets between Myr and psoriasis, Venny 2.1 software (https://bioinfogp.cnb.csic.es/tools/venny/index.html, accessed on 10 September 2025) was used to generate a Venn diagram by overlapping the active ingredient targets and psoriasis-related pathogenic targets. Subsequently, the intersection targets, Myr, and its active ingredients were imported into Cytoscape 3.10.0 software to construct the “Myr-Active Ingredients-Intersection Targets” network. This network was analyzed to explore the relationships between Myr, its active components, and the intersection targets. To further investigate the functional interactions of the intersection targets, the STRING database (https://string-db.org/, accessed on 10 September 2025) was used to obtain the protein–protein interaction (PPI) network of the intersection targets, with the protein type set to “Homo sapiens”. The resulting data was then imported into Cytoscape 3.10.0 to visualize the key genes in the PPI network. 

### 4.13. Statistical Analysis

Data are presented as mean ± SD and were analyzed using GraphPad Prism 9.0 (version 9.02) software. For comparisons among more than two groups, one-way ANOVA with Tukey’s post-test was used. A *p*-value of <0.05 was considered statistically significant.

## 5. Conclusions

Myr emerges as a multi-target natural compound capable of modulating EGFR-driven signaling and downstream inflammatory mediators. By bridging receptor-level modulation with broad immunoregulatory effects, it represents a promising candidate for psoriasis treatment, offering a mechanistic complement to current cytokine-focused therapies and highlighting the potential of natural products in complex inflammatory diseases.

## Data Availability

Data presented in this study is contained within the article.

## Abbreviations

The following abbreviations are used in this manuscript:

AKT	Protein kinase B
CBA	Cytometric bead array
DEX	Dexamethasone
DMSO	Dimethyl sulfoxide
EGFR	Epidermal growth factor receptor
FVS	Fixable viability stain
GAPDH	Glyceraldehyde-3-phosphate dehydrogenase
GO	Gene Ontology
HE	Hematoxylin and eosin staining
IHC	Immunohistochemistry
IFN-γ	Interferon gamma
IL	Interleukin
IMQ	Imiquimod
KEGG	Kyoto Encyclopedia of Genes and Genomes
MAPK	togen-activated protein kinase
MMP9	Matrix metalloproteinase 9
Myr	Myricetin
mTOR	Mammalian target of rapamycin
NF-κB	Nuclear factor kappa B
PASI	Psoriasis Area and Severity Index
PEG300	Polyethylene glycol 300
PI3K	Phosphatidylinositol 3-kinase
PPI	Protein–protein interaction
PTGS2	Prostaglandin–endoperoxide synthase 2
qPCR	Quantitative real-time polymerase chain reaction
TEWL	Transepidermal water loss
TNF	Tumor necrosis factor
